# Biologic Switching in Patients with Severe Asthma: Effects on Exacerbations, Corticosteroid Use and Lung Function

**DOI:** 10.3390/jcm15114149

**Published:** 2026-05-27

**Authors:** Fatma Dindar Çelik, Kurtuluş Aksu

**Affiliations:** Department of Immunology and Allergy, Ankara Ataturk Sanatorium Training and Research Hospital, University of Health Sciences, Ankara 06280, Türkiye; kurtulusaksu@yahoo.com

**Keywords:** asthma, asthma control, benralizumab, biologic therapy, corticosteroid use, exacerbation, mepolizumab, omalizumab, switching biologics

## Abstract

**Background/Objectives**: This study aimed to evaluate the clinical outcomes of biologic switching in patients with severe asthma in real-world settings. **Methods**: In this retrospective study, asthma exacerbations, systemic corticosteroid use, asthma control, and spirometric parameters were compared before and one year after biologic switching in adults with severe asthma. All patients were analyzed as a single cohort, with additional subgroup analyses performed according to the type of biologic switch (omalizumab to mepolizumab, mepolizumab to benralizumab, and omalizumab to benralizumab). **Results**: Among 29 patients who underwent biologic switching, the median annual exacerbation rate decreased from 2.0 (IQR, 1.5–2.5) to 0 (IQR, 0–1) at one year (*p* < 0.001), while systemic corticosteroid use declined from 69.0% (*n* = 20) to 34.5% (*n* = 10) (*p* = 0.002). A total of 38.0% of patients (*n* = 11) were switched from mepolizumab to benralizumab, 58.6% (*n* = 17) from omalizumab to mepolizumab, and 3.4% (*n* = 1) from omalizumab to benralizumab. In patients switched from omalizumab to mepolizumab, significant reductions were observed in annual exacerbations and in both the rate and dose of systemic corticosteroid use, along with improved asthma control (*p* = 0.002, *p* = 0.008, *p* = 0.002, and *p* = 0.001, respectively). In contrast, among patients switched from mepolizumab to benralizumab, exacerbation rates decreased significantly (*p* = 0.019), with no significant changes in systemic corticosteroid use (*p* = 1.000) or dose (*p* = 0.102). Only one patient was switched from omalizumab to benralizumab and showed improvements in exacerbation frequency, asthma control, systemic corticosteroid use, and spirometric parameters. The mean age of the patients was 53.24 ± 10.74 years (range, 33–73), with 51.7% being female (*n* = 15). **Conclusions**: Biologic switching was associated with favorable clinical outcomes, including fewer exacerbations and improved asthma control, in selected patients with severe asthma who showed an inadequate response to their current biologic therapy.

## 1. Introduction

Asthma affects more than 300 million individuals worldwide and can be effectively controlled in most patients with currently available inhaled therapies [1]. However, a subset remains difficult to manage despite optimized conventional treatment. This clinically heterogeneous group of patients with severe asthma may benefit from individualized biologic therapies. Biologic agents selectively target key molecular pathways involved in asthma pathogenesis. Mediators associated with type 2 inflammation, including interleukin-5 (IL-5), interleukin-4 (IL-4), interleukin-13 (IL-13), and immunoglobulin E (IgE), constitute the principal therapeutic targets of current biologic strategies [2].

Omalizumab (anti-IgE) was the first biologic approved for the treatment of asthma and has demonstrated efficacy in patients with allergic asthma [3]. Subsequently, therapies targeting eosinophilic inflammation were developed, including mepolizumab and reslizumab, which are anti-IL-5 monoclonal antibodies, and benralizumab, a monoclonal antibody targeting the IL-5 receptor α subunit (IL-5Rα), all of which have been shown to be effective in severe refractory eosinophilic asthma [4,5,6].

The selection of biologics in severe asthma requires an individualized and systematic clinical assessment. Decision-making should consider exacerbation burden, level of disease control under optimized therapy, and treatment-related adverse events. In addition, perennial aeroallergen sensitization, total IgE levels, and markers of eosinophilic inflammation are key determinants guiding appropriate biologic choice [7]. Following the initiation of biologic therapy, the Global Initiative for Asthma (GINA) recommends an initial 4-month trial period to evaluate treatment response; however, in selected patients, this period may be extended to 8–12 months [8]. The duration may be determined by local health authorities to prevent unnecessary use of healthcare resources, although in most cases the decision is left to the clinician’s individual judgment. If an adequate response is not achieved in terms of exacerbations, symptom control, lung function, reduction/discontinuation of oral corticosteroids, or if treatment-related adverse events occur, discontinuation of the biologic agent or switching to an alternative biologic may be considered in appropriate candidates.

In this study, we evaluated clinical outcomes associated with biologic switching, including asthma exacerbations, systemic corticosteroid use, asthma control, and spirometric parameters, in patients with uncontrolled asthma despite biologic therapy. Patients were further stratified by the type of biologic switch (omalizumab to mepolizumab, mepolizumab to benralizumab, and omalizumab to benralizumab) to evaluate differences in observed outcomes across switching patterns.

## 2. Materials and Methods

### 2.1. Study Design and Ethical Approval

This retrospective study was conducted at the Immunology and Allergy Department of a tertiary care hospital. The study adhered to the ethical principles of the Declaration of Helsinki and its subsequent amendments, and all procedures were performed in accordance with good clinical practice guidelines. Ethical approval was obtained from the local ethics committee (22 October 2025; BÇEK/391), and the requirement for written informed consent was waived due to the retrospective design of the study, which involved no direct patient contact and the use of previously recorded data.

### 2.2. Study Population and Data Collection

Patients with severe asthma who underwent switching of biologic agents between January 2015 and September 2025 due to inadequate asthma control and/or treatment-related adverse events were included in the study. Patients who did not meet the inclusion criteria, had a post-switch follow-up duration of less than 1 year for clinical outcome assessment, or had incomplete relevant clinical data were excluded. Given that omalizumab, mepolizumab, and benralizumab are reimbursed under the national healthcare system, patients who underwent switching among these three biologic agents were included in the study. Demographic and clinical data were obtained from patient records and the hospital information system.

Asthma control status, annual exacerbation rate, systemic corticosteroid use and dosage, and spirometric parameters including forced expiratory volume in one second (FEV1), forced vital capacity (FVC), and the FEV1/FVC ratio were recorded. Data obtained at the end of the previous biologic therapy (prior to switching) and at 1 year after initiation of the new biologic were compared in all patients. Patients were then grouped according to the type of biologic switch (omalizumab to mepolizumab, mepolizumab to benralizumab, and omalizumab to benralizumab) and analyzed.

All biologic agents were administered in accordance with approved dosing regimens. Mepolizumab (Nucala^®^, GlaxoSmithKline, Brentford, UK) was given at a dose of 100 mg subcutaneously every 4 weeks [9]. Omalizumab (Xolair^®^, Novartis Pharma AG, Basel, Switzerland) was administered subcutaneously every 2–4 weeks, with dosing based on baseline serum total IgE levels and body weight [10]. Benralizumab (Fasenra^®^, AstraZeneca, Cambridge, UK) was administered at a dose of 30 mg subcutaneously every 4 weeks for the first three doses, followed by every 8 weeks thereafter [11]. Switching between biologic agents was initiated directly after discontinuation of the previous agent.

Biologic therapies were initiated based on national reimbursement criteria, requiring severe uncontrolled asthma despite high-dose inhaled therapy and phenotype-specific eligibility. Omalizumab required evidence of allergic sensitization and total IgE levels within the dosing range (30–1500 IU/mL), while anti-IL-5/ IL-5Rα therapies required elevated blood eosinophil counts (≥150–300 cells/µL).

### 2.3. Definitions

Asthma control was assessed using the GINA assessment criteria [8]. According to the GINA, asthma control is evaluated based on four components over the preceding 4 weeks. Asthma was classified as well controlled when none of the following were present: daytime symptoms more than twice per week, night-time awakenings, reliever medication use more than twice per week, or any activity limitation; partially controlled when one or two of these features were present; and uncontrolled when three or four of these features were present.

Biologic therapy was switched in patients with treatment failure, defined as ≥2 exacerbations per year or at least one hospitalization, inability to taper or discontinue systemic corticosteroids, and inadequate asthma control.

Asthma exacerbations were defined as episodes of worsening asthma symptoms that required a short course of systemic corticosteroids and/or unscheduled healthcare utilization, including emergency department visits or hospitalizations. Systemic corticosteroid use referred to maintenance oral corticosteroid therapy.

### 2.4. Statistical Analysis

The data were analyzed using SPSS version 22.0 (IBM Corp., Armonk, NY, USA). The normality of data distribution was assessed using the Kolmogorov–Smirnov and Shapiro–Wilk tests. Continuous variables were presented as mean ± standard deviation (SD) for normally distributed data and as median (interquartile range [IQR]) for non-normally distributed data. Categorical variables were expressed as frequencies and percentages and compared using the chi-square test or Fisher’s exact test, as appropriate, while paired categorical variables were analyzed using McNemar’s test. Comparisons between independent groups were performed using the independent samples *t*-test for normally distributed variables and the Mann–Whitney U test for non-normally distributed variables. Paired comparisons were analyzed using the paired *t*-test for normally distributed variables and the Wilcoxon signed-rank test for non-normally distributed variables. A *p* value of <0.05 was considered statistically significant.

## 3. Results

A total of 34 patients who underwent biologic switching were evaluated. Of these, five were excluded. Two patients with an eosinophilic phenotype suitable for mepolizumab had initially received off-label omalizumab because mepolizumab was not available in the country at that time. After mepolizumab became covered by the national reimbursement system, these patients were switched from omalizumab to mepolizumab for reimbursement-related reasons rather than treatment failure or poor asthma control; therefore, they were excluded. One patient was switched from mepolizumab to omalizumab because of concomitant urticaria despite well-controlled asthma and was excluded because the switch was not driven by poor asthma control. Two additional patients were excluded because switching had occurred within the previous three months, which was considered early for assessing its effect on asthma control. The final study population comprised 29 patients with severe asthma, with a mean age of 53.24 ± 10.74 years (range, 33–73). The patients comprised 51.7% females (*n* = 15) and 48.3% males (*n* = 14), with a median asthma duration of 18 years (IQR, 12–25.5). Demographic and clinical characteristics of the study population are summarized in Table 1.

Before switching biologic therapy, 62.1% (*n* = 18) of patients were treated with omalizumab and 37.9% (*n* = 11) with mepolizumab. Mepolizumab was initiated in 58.6% (*n* = 17) of patients and benralizumab in 41.4% (*n* = 12). A total of 38.0% of patients (*n* = 11) were switched from mepolizumab to benralizumab, 58.6% (*n* = 17) from omalizumab to mepolizumab, and 3.4% (*n* = 1) from omalizumab to benralizumab. Adverse events were observed in 6.9% (*n* = 2) of patients: headache in one patient receiving omalizumab and oral mucosal lesions in another receiving mepolizumab.

The median duration of biologic therapy prior to switch among all patients was 32 months (IQR, 8–54.5). In the omalizumab to mepolizumab group, the median duration of omalizumab treatment was 40 months (IQR, 5–62), whereas in the mepolizumab to benralizumab group, the median duration of mepolizumab treatment was 32 months (IQR, 10–40). The single patient who switched from omalizumab to benralizumab had received omalizumab for 12 months before switching. The median annual exacerbation rate decreased from 2.0 (IQR, 1.5–2.5) before the biologic switch to 0 (IQR, 0–1) at 1 year after switching, representing a statistically significant reduction (*p* < 0.001). Systemic corticosteroids were used by 69.0% (*n* = 20) of patients prior to the biologic switch, which decreased to 34.5% (*n* = 10) at one year after switching (*p* = 0.002). Among the 20 patients receiving systemic corticosteroids, treatment was discontinued in 50% (*n* = 10), while the dose was reduced in 25% (*n* = 5). Only one patient (3.8%) among all patients discontinued the biologic initiated after the switch due to an inadequate treatment response, and this patient was in the group switched from mepolizumab to benralizumab.

When the groups switched from omalizumab to mepolizumab and from mepolizumab to benralizumab were compared, no significant differences were observed between the two groups in terms of age, sex, aeroallergen sensitization, body mass index, asthma duration, total IgE levels, smoking status, presence of chronic rhinosinusitis with nasal polyps, systemic corticosteroid use, high- or medium-dose inhaled corticosteroid–long-acting β2-agonist (ICS–LABA) therapy, or long-acting muscarinic antagonist (LAMA) use (*p* > 0.05 for all comparisons). The eosinophil count was significantly lower in the group switched from mepolizumab to benralizumab (*p* = 0.013); additionally, montelukast use was more frequent in this group (*p* = 0.019). As only one patient was switched from omalizumab to benralizumab, no comparative analysis with the other groups could be performed.

When all patients were evaluated, significant differences in FEV1 (mL), FVC (mL), and FVC (%) were observed between pre-switch values and those at one year after switching (*p* = 0.024, 0.003, and 0.047, respectively). However, in the group switched from omalizumab to mepolizumab, a significant difference was observed only in FVC (%) (*p* = 0.036) and in the group switched from mepolizumab to benralizumab, significant differences were observed in FEV1 (mL) and FVC (mL) (*p* = 0.034 and *p* = 0.027, respectively) (Table 2).

In the group switched from omalizumab to mepolizumab, significant reductions in exacerbation rates and in the rate and dose of systemic corticosteroid use were observed at the end of the first year, along with a significant improvement in asthma control (*p* = 0.002, *p* = 0.008, *p* = 0.002, and *p* = 0.001, respectively). In the group switched from mepolizumab to benralizumab, a significant reduction in exacerbations was observed (*p* = 0.019); however, no statistically significant changes were found in the number of patients receiving systemic corticosteroids (*p* = 1.000) or in their dosage (*p* = 0.102). In this group, McNemar–Bowker test could not be computed due to sparse data and the presence of zero cells in the contingency table. A shift toward well-controlled asthma was observed after switching to benralizumab; however, statistical significance could not be assessed.

In both groups, a reduction was observed in the number of patients receiving high-dose ICS–LABA therapy; however, this change did not reach statistical significance in either the omalizumab to mepolizumab group (*p* = 0.063) or the mepolizumab to benralizumab group (*p* = 0.250).

As only one patient (3.4%) was switched from omalizumab to benralizumab. Under omalizumab treatment, the patient experienced one exacerbation that year and was receiving 8 mg/day of maintenance methylprednisolone. During the first year after switching to benralizumab, the patient remained exacerbation-free and systemic corticosteroids were discontinued. Improvement in spirometric parameters was also observed, as shown in Table 3.

## 4. Discussion

Based on the analysis of data from all patients included in the study, switching biologic therapy was associated with a significant reduction in the annual asthma exacerbation rate, improvement in asthma control, and decreases in both the number of patients requiring systemic corticosteroids and the corresponding doses. Although improvements were observed in spirometric parameters, not all changes reached statistical significance. When patients were stratified according to switches across different pathways (omalizumab to mepolizumab) and switches within the same pathway to a different receptor target (mepolizumab to benralizumab), a significant reduction in asthma exacerbations was observed in both groups. However, a significant reduction in corticosteroid use was observed in patients who switched to a different pathway, whereas no statistically significant reduction was detected in those who underwent a switch within the same pathway.

Biologic agents have been demonstrated in clinical trials to reduce asthma exacerbations and systemic corticosteroid use in patients with severe asthma [5,12,13]. Nevertheless, a subset of patients may have an inadequate response to biologic therapy. In such cases, switching between biologic agents may be considered a strategy to optimize asthma control. In our study, the association between biologic switching and clinical outcomes, including asthma exacerbations, disease control, and systemic corticosteroid use, was evaluated using real-world data rather than data obtained under the restrictive conditions of clinical trials. The clinical characteristics of all patients who underwent a biologic switch were evaluated; additionally, patients who switched from omalizumab to mepolizumab and from mepolizumab to benralizumab were analyzed as separate subgroups. As only one patient was switched from omalizumab to benralizumab, this patient was presented descriptively as a single case, and statistical comparisons with the other two groups were not performed. There were no statistically significant differences between the two groups in terms of clinical characteristics, except for eosinophil levels and montelukast use (Table 1). Eosinophil levels were lower in the group in which mepolizumab was discontinued, likely reflecting the eosinophil-lowering effect of prior mepolizumab treatment [14]. Nevertheless, given the small subgroup sizes, the absence of statistical significance in other baseline variables should be interpreted cautiously rather than as definitive evidence of baseline comparability.

Because omalizumab was the first biologic approved for severe asthma, reports on switching from omalizumab to other biologic agents are more available in the literature. In a clinical trial conducted by Chapman et al., patients with uncontrolled severe eosinophilic asthma who were switched directly from omalizumab to mepolizumab showed clinically meaningful improvements in asthma control, health-related quality of life, and exacerbation frequency, without any safety or tolerability concerns [15]. In a report presenting post hoc analyses of the MENSA and SIRIUS trials evaluating the effect of mepolizumab in patients with severe eosinophilic asthma previously treated with omalizumab, the MENSA study showed that mepolizumab reduced exacerbation rates by 57% in patients with prior omalizumab use and by 47% in those without prior omalizumab use, compared with placebo. In addition, the SIRIUS study demonstrated a reduction in oral corticosteroid use, and this reduction was found to be independent of prior omalizumab use [16]. In a single-center real-world study including 10 patients who were switched from omalizumab to anti-IL-5 therapy (benralizumab or mepolizumab), a significant reduction in annual exacerbation rates was observed, whereas the reduction in systemic corticosteroid dose did not reach statistical significance [17]. In another study, a retrospective, multicenter analysis including 27 patients reported that switching from omalizumab to mepolizumab was associated with a reduction in annual asthma exacerbations, along with decreases in systemic corticosteroid use and dose, and an improvement in asthma control [18]. In a study evaluating data from the pre-biologic period, during omalizumab treatment, and after switching to mepolizumab, the mean annual number of exacerbations decreased from 5.8 ± 1.8 in the 12 months prior to biologic therapy to 4.2 ± 1.4 following omalizumab treatment, and further to 0.7 ± 0.9 after mepolizumab therapy. This reduction with mepolizumab was statistically significant compared with both the pre-treatment period and the omalizumab treatment phase (*p* < 0.001), and a significant reduction in daily oral corticosteroid use was also reported [19]. In our study, patients who were switched from omalizumab to mepolizumab, a statistically significant reduction was observed in the annual asthma exacerbation rate, along with decreases in systemic corticosteroid use and dosage, and a concomitant improvement in asthma control. Our findings are consistent with previous studies reporting that switching from omalizumab to mepolizumab can lead to clinically meaningful improvements in exacerbation rates and corticosteroid burden. This favorable clinical pattern observed after switching may reflect, at least in part, the relevance of IL-5 mediated eosinophilic inflammation in patients with severe asthma who remain uncontrolled despite anti-IgE therapy. However, given the retrospective pre-post design and the absence of a non-switching control group, these improvements may not be definitively attributed to the biologic switch itself. From a clinical perspective, our findings suggest that switching to IL-5 targeted biologic therapy may be considered in selected patients with inadequate response to anti-IgE therapy, particularly in the presence of eosinophilic inflammation.

When spirometric parameters in patients switched from omalizumab to mepolizumab were evaluated, a real-life study showed significant increases in FEV1 (L), FEV1 (%), and FEV_1_/FVC, but not in FVC (L) or FVC (%) [18]. In another study examining spirometric changes alongside asthma exacerbation rates and asthma control, although a clinically meaningful increase in FEV1 (>100 mL) was observed, this improvement was reported to be more limited compared with the improvements seen in other parameters [15]. In our study, although improvements were observed in spirometric parameters in patients switched from omalizumab to mepolizumab, only the increase in FVC (%) reached statistical significance. Overall, switching from omalizumab to mepolizumab was associated with reduced asthma exacerbations, decreased corticosteroid use, and significant improvement in asthma control, whereas changes in spirometric parameters were more limited. This finding may reflect the fact that spirometric measurements do not always fully capture changes in patient-reported symptoms and asthma control. In addition, long-standing severe asthma may be associated with airway remodeling, which could limit the extent of lung function improvement observed after treatment modification.

The current evidence base for switching from mepolizumab to benralizumab is limited and predominantly consists of retrospective, real-world studies. In a retrospective study by Kavanagh et al. [20] including 33 patients with severe eosinophilic asthma who were switched from mepolizumab to benralizumab, the annual asthma exacerbation rate decreased from 3.89 (±2.17) during mepolizumab treatment to 1.64 (±1.57) at week 48 of benralizumab, and this reduction was statistically significant (*p* < 0.001) [20]. In the same study, although a decrease in the number of patients requiring systemic corticosteroids was observed when comparing the end of mepolizumab treatment with week 48 of benralizumab, this change did not reach statistical significance (*p* = 0.090) [20]. In another real-world study by Numata et al., including 24 patients receiving benralizumab, 11 patients had been switched from mepolizumab. In this switched subgroup, no significant improvement was observed in the annual asthma exacerbation rate, systemic corticosteroid dose, or asthma control at the median 14-month follow-up compared with baseline [21]. In a multicenter study conducted by Drick et al., patients who were switched from anti-IL-5 therapy (mepolizumab or reslizumab) to anti-IL-5Rα therapy (benralizumab) were evaluated [22]. In these patients, annual asthma exacerbation rates prior to biologic therapy were compared with those observed during anti-IL-5 and anti-IL-5Rα treatment. Although a significant reduction was observed compared with the pre-biologic baseline, no significant difference in annual exacerbation rates was found when comparing values during anti-IL-5 therapy and after the switch to anti-IL-5Rα therapy. However, a significant reduction was observed in both the number of patients requiring systemic corticosteroids and the corticosteroid dose following the switch. As observed, the available literature presents heterogeneous findings regarding the effects of switching from mepolizumab to benralizumab on asthma exacerbations and corticosteroid use, which may be attributable to variability in patient characteristics in real-world settings. Our findings demonstrated a significant reduction in asthma exacerbation rates in patients switched from mepolizumab to benralizumab; although decreases were observed in both the number of patients requiring systemic corticosteroids and their doses, these changes did not reach statistical significance. The lack of a significant association between switching from mepolizumab to benralizumab and reduced corticosteroid use may reflect the partial suppression of eosinophilic inflammation already achieved with prior anti-IL-5 therapy. However, the distinct mechanism of benralizumab, which induces near-complete eosinophil depletion through IL-5Rα blockade, may still provide additional benefit in reducing exacerbations. Therefore, further large-scale studies are warranted to better elucidate the impact of switching biologics within the same pathway on asthma control and treatment outcomes.

Regarding spirometric changes, a real-life study by Numata et al. reported no significant improvements in spirometric parameters among patients who were switched from mepolizumab to benralizumab [21]. In a multicenter study assessing patients switched to benralizumab from omalizumab or mepolizumab, a subgroup of 24 patients previously treated with mepolizumab was analyzed. At 12 months, significant improvements were observed in asthma control and the annual asthma exacerbation rate; however, the increase in FEV1 did not reach statistical significance [23]. In another study, FEV1 (mL) and FEV1 (%) were significantly increased at week 48 of benralizumab compared with the end of mepolizumab treatment [20]. In our study, significant improvements were observed in FEV1 (mL) and FVC (mL), while increases in other spirometric parameters did not reach statistical significance. The improvement in absolute lung volumes may reflect a reduction in airway inflammation and air trapping after benralizumab therapy. In contrast, the lack of significant change in percentage-predicted parameters may be related to the relatively small sample size. Considering the sample sizes of the switching subgroups, these findings were interpreted as exploratory clinical observations rather than definitive comparative conclusions.

Among the study population, only one patient was switched from omalizumab to benralizumab. In this patient, no exacerbations were observed during the one-year follow-up, systemic corticosteroid therapy was discontinued, and better asthma control was achieved. In addition, spirometric parameters showed improvement (Table 3). However, as this subgroup included only a single patient, no statistical analysis could be performed.

A key strength of our study is that it evaluates not only switches from the anti-IgE pathway to the anti-IL-5 pathway, but also transitions between biologic agents targeting different receptors within the anti-IL-5 pathway, a dimension that has been less extensively addressed in the existing literature. This study has several limitations. First, its retrospective design restricted the availability and scope of additional data for analysis. Second, the absence of a non-switching control group limits the ability to establish a causal relationship between biologic switching and the observed clinical improvements. Third, the relatively small sample size may limit the generalizability of the findings.

In conclusion, in this retrospective cohort, biologic switching was associated with reductions in asthma exacerbations and improvements in symptom control among selected patients with severe asthma who had an inadequate response to prior biologic therapy. Further large-scale controlled studies are needed to better clarify the clinical outcomes associated with biologic switching and to define its role in the management of severe asthma.

## Figures and Tables

**Table 1:** Demographic and clinical characteristics of the patients.

Variables	All Patients (*n* = 29)	Omalizumab → Mepolizumab (*n* = 17)	Mepolizumab → Benralizumab (*n* = 11)	*p* Value
Age (years)	53.24 ± 10.74	53.52 ± 10.25	53.82 ± 11.89	0.946
Female gender; *n* (%)	15 (51.7%)	10 (58.8%)	5 (45.5%)	0.488
Presence of aeroallergen sensitization; (%)	18 (62.1%)	12 (70.6%)	6 (54.5%)	0.387
Asthma duration (years), median values (IQR)	18.0 (12.0–25.5)	16.0 (12.0–25.5)	18.0 (11.0–25.0)	0.777
BMI (kg/m^2^)	29.06 ± 4.77	28.90 ± 5.39	29.58 ± 4.02	0.720
Eosinophil count (10^6^/L), median values (IQR)	470.0 (295–920)	680 (440–1110)	360 (130–450)	0.013
IgE (kIU/L), median values (IQR)	180 (37.5–460.5)	238 (72.5–493.5)	75 (29–240)	0.188
Current smoker; *n* (%)	2 (6.9%)	1 (5.9%)	1 (9.1%)	0.747
Concomittant nasal polyps	14 (48.3%)	9 (52.9%)	5 (45.5%)	0.699
Medications				
Systemic corticosteroids	20 (69.0%)	12 (70.6%)	7 (63.6%)	0.700
Medium-dose ICS+LABA	10 (34.5%)	7 (41.2%)	3 (27.3%)	0.453
High-dose ICS+LABA	19 (65.5%)	10 (58.8%)	8 (72.7%)	
LAMA	25 (86.2%)	14 (82.4%)	10 (90.9%)	0.527
Montelukast	13 (44.8%)	4 (23.5%)	8 (72.7%)	0.019

Data are summarized as mean ± standard deviation unless otherwise stated. BMI, Body mass index; ICS: Inhaled corticosteroid; IQR, Interquartile range; LABA, Long-acting β2-agonist; LAMA, Long-acting antimuscarinic agent.

**Table 2:** Pre-switch values and 1-year outcomes of all patients and subgroups according to biologic switch type.

Variables	All Patients (*n* = 29)			Omalizumab → Mepolizumab (*n* = 17)			Mepolizumab → Benralizumab (*n* = 11)		
	Prior to Switch	Year 1	*p* Value	Prior to Switch	Year 1	*p* Value	Prior to Switch	Year 1	*p* Value
Exacerbations, median values (IQR)	2 (1.5–2.5)	0 (0–1)	<0.001	2 (1.5–2)	0 (0–1)	0.002	2 (2–4)	0 (0–1)	0.019
Systemic corticosteroid ^a^ usage; *n* (%)	20 (69.0%)	10 (34.5%)	0.002	12 (70.6%)	4 (23.5%)	0.008	7 (63.6%)	6 (54.5%)	1.000
Systemic corticosteroid ^a^ dose (mg), median values (IQR)	8 (4–8)	1 (0–4)	<0.001	6 (4–8)	0 (0–2)	0.002	8 (4–16)	4 (4–8)	0.102
ICS; *n* (%) Medium-dose ICS+LABA High-dose ICS+LABA	10 (34.5%) 19 (65.5)	18 (62.1%) 11 (37.9%)	0.008	7 (41.8%) 10 (58.8%)	12 (70.6%) 5 (29.4%)	0.063	3 (27.3%) 8 (72.7%)	6 (54.5%) 5 (45.5%)	0.250
Asthma control status; *n* (%) Well controlled Partly controlled Uncontrolled	1 (3.4%) 7 (24.1%) 21 (72.4%)	25 (86.2%) 2 (6.9%) 2 (6.9%)	<0.001	1 (5.9%) 4 (23.5%) 12 (70.6%)	15 (88.2%) 1 (5.9%) 1 (5.9%)	0.001	0 3 (27.3%) 8 (72.7%)	9 (81.1%) 1 (9.1%) 1 (9.1%)	^b^
FEV1 (mL)	2098.28 ± 854.76	2250.00 ± 848.98	0.025	2180.58 ± 839.01	2252.35 ± 792.24	0.406	1842.72 ± 797.97	2081 ± 809.44	0.034
FEV1 (%)	68.65 ± 21.73	73.17 ± 19.90	0.064	72.88 ± 20.79	76.29 ± 21.40	0.233	60.63 ± 22.41	66.55 ± 16.50	0.229
FVC (mL)	2807.24 ± 1100.43	3024.83 ± 1156.77	0.003	2794.12 ± 862.90	2928.82 ± 861.35	0.117	2556.36 ± 1113.60	2846.36 ± 1102.91	0.027
FVC (%)	74.28 ± 19.16	78.31 ± 17.21	0.047	76.59 ± 14.60	80.94 ± 16.13	0.036	67.00 ± 21.30	71.18 ± 15.24	0.352
FEV1/FVC	74.38 ± 10.38	74.82 ± 10.27	0.685	76.41 ± 9.79	76.47 ± 10.67	0.972	72.45 ± 10.91	73.54 ± 9.36	0.447

Data are summarized as mean ± standard deviation (minimum–maximum) unless otherwise stated. ^a^ Methylprednisolone equivalent; ^b^ McNemar–Bowker test could not be performed due to sparse data and zero cell counts in the contingency table. A shift toward well-controlled asthma was observed after switching to benralizumab; however, statistical significance could not be assessed. FEV1, forced expiratory volume in the 1st second; FVC, forced vital capacity; ICS: Inhaled corticosteroid; IQR, Interquartile range; LABA, Long-acting β2-agonist; LAMA, Long-acting antimuscarinic agent.

**Table 3:** Pre-switch values and 1-year clinical outcomes of the patient switched from omalizumab to benralizumab.

Variables	Omalizumab → Benralizumab (*n* = 1)	
	Prior to Switch	Year 1
Exacerbations,	1	0
Systemic corticosteroid Usage	Yes	No
Systemic corticosteroid dose (mg) ^a^	8	0
ICS	High-dose ICS+LABA	High-dose ICS+LABA
Asthma control status	Uncontrolled	Well controlled
FEV1 (mL)	3520	4060
FEV1 (%)	85	93
FVC (mL)	5790	6620
FVC (%)	115	112
FEV1/FVC	61	61

^a^ Methylprednisolone equivalent; FEV1, forced expiratory volume in the 1st second; FVC, forced vital capacity; ICS: Inhaled corticosteroid; LABA, Long-acting β2-agonist.

## Data Availability

The data set used and/or analyzed during the present study is available upon reasonable request.

## Abbreviations

The following abbreviations are used in this manuscript:

FEV1	Forced expiratory volume in one second
FVC	Forced vital capacity
GINA	Global Initiative for Asthma
ICS	Inhaled corticosteroid
IgE	Immunoglobulin E
IL-4	Interleukin-4
IL-5	Interleukin-5
IL-5Rα	Interleukin-5 receptor α
IL-13	Interleukin-13
LABA	Long-acting β2-agonist
LAMA	Long-acting muscarinic antagonist
